# Thermoresistive Properties of Graphite Platelet Films Supported by Different Substrates

**DOI:** 10.3390/ma12213638

**Published:** 2019-11-05

**Authors:** Mariano Palomba, Gianfranco Carotenuto, Angela Longo, Andrea Sorrentino, Antonio Di Bartolomeo, Laura Iemmo, Francesca Urban, Filippo Giubileo, Gianni Barucca, Massimo Rovere, Alberto Tagliaferro, Giuseppina Ambrosone, Ubaldo Coscia

**Affiliations:** 1 Institute for Polymers, Composites and Biomaterials—National Research Council (IPCB-CNR). SS Napoli/Portici, Piazzale E. Fermi, 1-80055 Portici (NA), Italy; mariano.palomba@cnr.it (M.P.); giancaro@unina.it (G.C.); andrea.sorrentino@cnr.it (A.S.); 2 Department of Physics ‘E.R.Caianello’, University of Salerno, Via Giovanni Paolo II, 132—84084 Fisciano (SA), Italy; liemmo@unisa.it (L.I.); furban@unisa.it (F.U.); 3 Superconducting and Other Innovative Materials and Devices Institute—National Research Council (SPIN-CNR), Via Giovanni Paolo II, 132—84084 Fisciano (SA), Italy; filippo.giubileo@spin.cnr.it; 4 Department SIMAU, Polytechnic University of Marche, Via Brecce Bianche, I-60131 Ancona, Italy; g.barucca@staff.univpm.it; 5 Department of Applied Science and Technology, Politecnico di Torino. Corso Duca degli Abruzzi, 24, 10129 Torino, Italy; massimo.rovere@polito.it (M.R.); alberto.tagliaferro@polito.it (A.T.); 6 Department of Physics ‘Ettore Pancini’, University of Naples ‘Federico II’, Via Cintia, I-80126 Napoli, Italy; giuseppina.ambrosone@unina.it (G.A.); coscia@fisica.unina.it (U.C.); 7 CNISM, Naples Unit, Via Cintia, I-80126 Napoli, Italy

**Keywords:** graphite platelet coatings, LDPE, thermal expansion coefficient, thermoresistive properties

## Abstract

Large-area graphitic films, produced by an advantageous technique based on spraying a graphite lacquer on glass and low-density polyethylene (LDPE) substrates were studied for their thermoresistive applications. The spray technique uniformly covered the surface of the substrate by graphite platelet (GP) unities, which have a tendency to align parallel to the interfacial plane. Transmission electron microscopy analysis showed that the deposited films were composed of overlapped graphite platelets of different thickness, ranging from a few tens to hundreds of graphene layers, and Raman measurements provided evidence for a good graphitic quality of the material. The GP films deposited on glass and LDPE substrates exhibited different thermoresistive properties during cooling–heating cycles in the −40 to +40 °C range. Indeed, negative values of the temperature coefficient of resistance, ranging from −4 × 10^−4^ to −7 × 10^−4^ °C^−1^ have been observed on glass substrates, while positive values varying between 4 × 10^−3^ and 8 × 10^−3^ °C^−1^ were measured when the films were supported by LDPE. These behaviors were attributed to the different thermal expansion coefficients of the substrates. The appreciable thermoresistive properties of the graphite platelet films on LDPE could be useful for plastic electronic applications.

## 1. Introduction

Plastic electronics is an emerging technological field with a remarkable potential in the areas of robotics, solar energy, sensors, health care, industrial automation, etc. [1,2,3,4,5]. Plastic electronic devices offer the unique characteristics of stretchability, flexibility, transparency, lightweightness, etc., which can be exploited for future industrial applications. Additionally, the processing technologies applied for the fabrication of plastic electronic devices (e.g., contact printing, roll-to-roll, ink-jet, spraying, etc.) are inexpensive and powerful, compared to the traditional approaches available for silicon-based electronics. However, all these technologies require further optimization to allow the production of these materials on a large scale.

In this field, plastics are useful both for fabricating printed circuit boards and for making the active and passive electronic components of a circuit. These components can be easily achieved by incorporating functional organic materials (e.g., chromophores, fluorophores, conductive or magnetic fillers, etc.) into an adequate polymer matrix [6,7,8]. In particular, graphite platelets, carbon nanotubes, fullerene, graphene, and other carbonaceous materials have been extensively studied and utilized to obtain conductive, thermoresistive, and semiconductive polymeric nanocomposites [9,10,11,12,13,14,15,16,17]. Furthermore, the surfaces of polymers such as poly (methyl methacrylate), polyethylene terephthalate and low-density polyethylene (LDPE) have been made conductive by depositing graphite or graphene layers onto them for the fabrication of printed radio frequency devices [18], electrically conductive paths [19], piezoresistive sensors [20], and strain gauges [9]. These layers can be deposited by chemical vapor deposition [21], casting and drying inks [18], micromechanical techniques based on spreading an alcoholic suspension of graphite nanoplatelets [22,23] and spraying conductive composites [9]. In particular, this last technique is easy, inexpensive, and industrially scalable for the fabrication of large area films.

In this study, the properties of graphite platelet (GP) films, obtained by spraying a commercial lacquer on different substrates (LDPE and glass), were investigated. The deposited coatings were morphologically and structurally characterized by scanning electron microscopy (SEM), transmission electron microscopy (TEM), Fourier-transform infrared spectroscopy (FT-IR), and Raman spectroscopy. The thermal properties of the commercial lacquer, pure LDPE and graphite platelets deposited on LDPE were also explored by thermogravimetric analysis (TGA). The thermal expansion coefficients of the LDPE substrates coated by GP films were determined by dynamic–mechanical thermal analysis (DMTA). Thermoresistive measurements of graphite platelets films on glass and LDPE substrates, i.e., the measurements of the electrical resistance as a function of the temperature, were carried out during the cooling–heating cycles between −40 °C and +40 °C. Owing to the thermal expansion coefficient mismatch, the substrate could dramatically affect the temperature coefficient of resistance (TCR) of the GP film, which exhibited a negative TCR on glass and a positive TCR on the LDPE substrate.

## 2. Materials and Methods

Large area thin films of graphite-based material were deposited on glass and LDPE substrates by spray technology, using a commercial lacquer, Graphit 33 (from Kontakt Chemie, Zele, Belgium), which is commonly used in optical and electrical fields [24]. In order to produce a full cone jet spot, the spray nozzle was horizontally directed, taking it at a distance of 20 cm from the substrate surface. After spraying, the coated substrates were dried in air at room temperature, for 4 h.

Scanning electron microscopy analysis of the sample surface was performed using a FEI Quanta 200 FEG (FEI, Hillsboro, Oregon, USA) microscope. Due to the conductive nature of the graphite-based material, samples were observed without any preparation except for the electrical grounding of the surface. The inner structure of the deposited material was investigated through transmission electron microscopy measurements carried out by a Philips CM200 (Philips, Amsterdam, The Netherlands) microscope, operating at 200 kV and equipped with a LaB_6_ filament. For TEM observations, two kinds of samples were prepared. In one case, the Graphit 33 was sprayed in acetone, the solid phase was isolated by centrifugation and deposited on a TEM copper grid covered with a thin carbon film. In another case, in order to see the inner structure of the deposited layer the Graphit 33 was sprayed on a substrate and prepared in a cross-section by the conventional thinning procedure, consisting of mechanical polishing by grinding papers, diamond pastes, and a dimple grinder; final thinning was carried out by an ion beam system (Gatan PIPS), using Ar ions at 5 kV.

Fourier-transform infrared spectra of dry Graphit 33 were made by a MIR/FIR Spectrometer (Frontier, PerkinElmer, Milan, Italy). The samples preparation was performed by mixing powered Graphit 33 with a crystalline KBr powder in an adequate ratio (1% by weight) and this mixture was cold pressed under vacuum at 8 tons, for 10 min, to obtain a transparent pellet.

The thermal gravimetric analysis, in the 40–600 °C range was used to establish the thermal stability of the coating, the pure LDPE, and the LDPE coated by the GP film. Such analyses were performed by a TA-Instrument (Q500, Milan, Italy), operating in flowing nitrogen, with a constant heating rate of 10 °C/min.

Raman spectra of GP films on glass and LDPE substrates were performed by a Raman spectrometer InviaH-Renishaw (InviaH, Renishaw, plc, New Mils, Wotton-under Edge, Glowcester Shire, GL128JR, UK). A green argon laser with a 514.5 nm wavelength and a beam size approximately 2 μm in diameter was selected for the analysis. A microscope with a 50X magnification power was used with an exposure time of 10 s. Extended scans from 100 cm^−1^ to 3500 cm^−1^ was performed using a laser power of ca. 5 mW (5% of available laser power). An in-house MATLAB software (Matlab 9.7.0.1190202 R2019b, Mathworks Inc., Natick, MA, USA) was used to correct the quantum efficiency of the detector, conduct the baseline subtraction, and carry out the data processing.

Thermal expansion and contraction tests of an LDPE sample coated by GP (18.7 × 8.9 mm and ca. 90 µm thick) was carried out by means of cooling–heating cycles in the −40 to +40 °C range, at a rate of 5 °C/min, using a thermal mechanical analyzer (TMA2940, TA-Instruments, New Castle, USA).

Electrical measurements were executed under vacuum (∼2 mbar) in a coplanar configuration by silver paint contacts (1-cm long and 1-mm spaced) spread on their surfaces. Vacuum was adopted as a precaution to avoid ice formation during the low temperature cycle, as well as to prevent possible effects of moisture or other adsorbates. Current–voltage (I–V) characteristics were taken in a Janis Research ST-500 probe station equipped (Janis Research, Woburn, MA, USA) with 4 micromanipulators connected to a Source-Measurement Unit (SMU) Keithley 4200-SCS (Tektronix, Inc., Beaverton, OR, USA). From each I–V and from monitoring the resistance during a period of 60 s it was estimated that the mean resistance of the samples at different temperatures T during cooling–heating cycles from −40 °C to +40 °C performed at a rate of about 5 °C/min.

## 3. Results and Discussion

### 3.1. Characterization of Graphit 33 Lacquer

**Figure 1:**
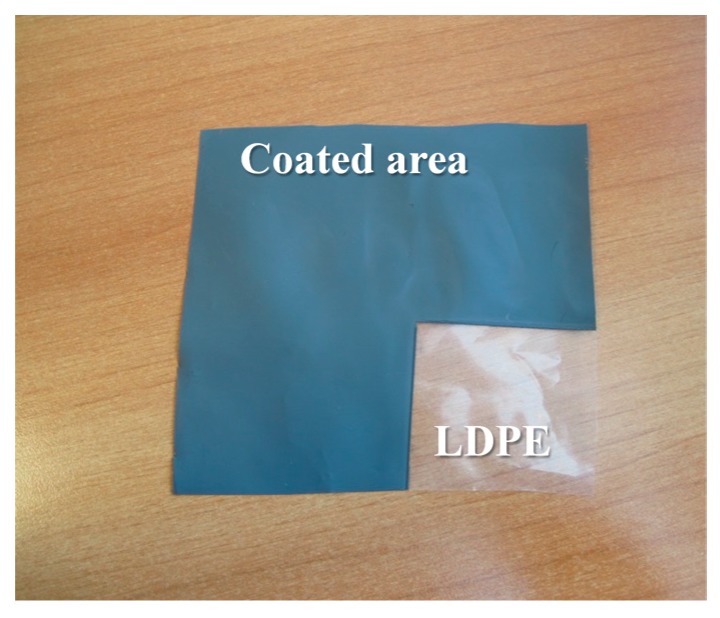
Large-area film after spraying Graphit 33 lacquer onto the low-density polyethylene (LDPE) film.

Large-area thin films of graphite-based material were deposited onto the glass and LDPE substrates through spray technology, using a commercial lacquer such as Graphit 33 (see Figure 1).

**Figure 2:**
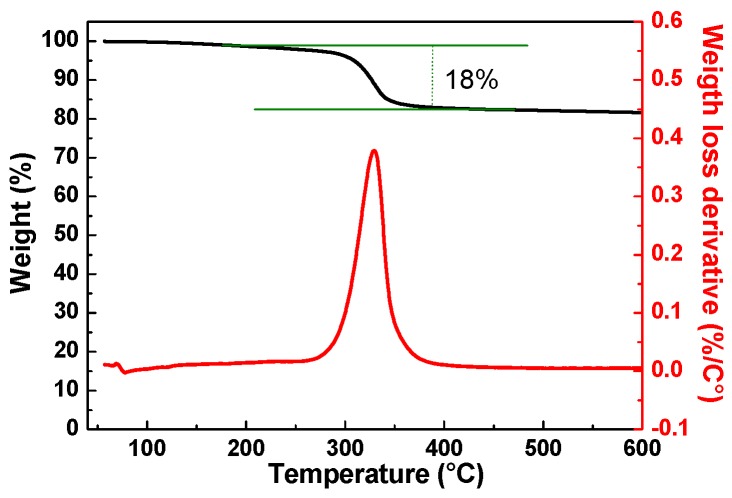
TGA–thermogram and derivative thermogravimetric plot profile of a typical dried Graphit 33 sample.

Thermo-gravimetric analysis and infrared spectroscopy were carried out to identify the composition and concentration of the lacquer. The TGA–thermogram of a dry Graphit 33 sample is shown in Figure 2. The sample is characterized by a weight loss in the 250–400 °C range, with a maximum degradation rate at ca. 330 °C, as can be seen from the derivative curve (red line in Figure 2). This weight loss could be attributed to the thermal degradation of the polymeric binder contained in the product, and it was estimated as ca. 18% by weight. As evidenced by the thermogram, the absence of weight loss at low temperature confirmed the absence of a volatile solvent after drying.

**Figure 3:**
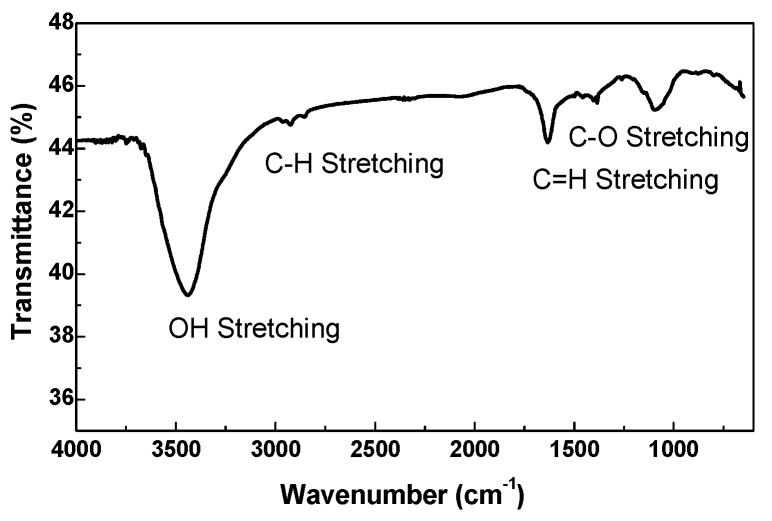
FT–IR spectrum of a typical dried Graphit 33 sample in KBr.

The FT–IR spectrum of the dry Graphit 33 in KBr is shown in Figure 3. The main absorption bands were centered at 3440 cm^−1^ (–OH group stretching), 2927 cm^−1^ (C–H group stretching), 1631 cm^−1^ (C=C group stretching), and 1092 cm^−1^ (C–O group stretching).

**Figure 4:**
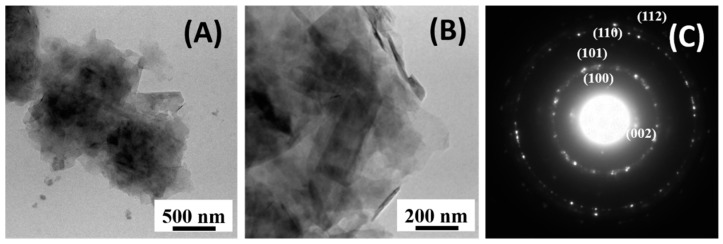
Graphite platelets extracted by the Graphit 33 lacquer: (**A**,**B**) bright field TEM images taken at different magnifications; and (**C**) corresponding SAED pattern in which the diffraction rings can be associated with the families of graphite atomic planes.

TEM measurements were carried out to investigate the inner structure of the graphite phase. In particular, Figure 4A,B show the solid phase extracted by the Graphit 33 by using acetone to remove the polymeric binder. It was evident that the filler was made of platelets that in the images appear to be largely superimposed. The platelets were hundreds of nanometers large, and their thicknesses were quite small, considering the low contrast visible in the images. Selected area electron diffraction (SAED) measurements were used to investigate the phase of the filler. Figure 4C shows a typical SAED pattern. All the diffraction rings could be associated with the pure graphite phase (International Centre for Diffraction Data, ICDD, card n^o^. 41–1487) confirming that the only crystalline phase inside the Graphit 33 was graphite.

All experimental observations shown so far reveal that the Graphit 33 lacquer is composed mainly of graphite platelets. The presence of the binder allows us to deposit a continuous graphite coating on several types of substrates (glass, silicon, LDPE, etc.) using spray technology.

### 3.2. Morphological and Structural Characterizations of the GP Coatings

**Figure 5:**
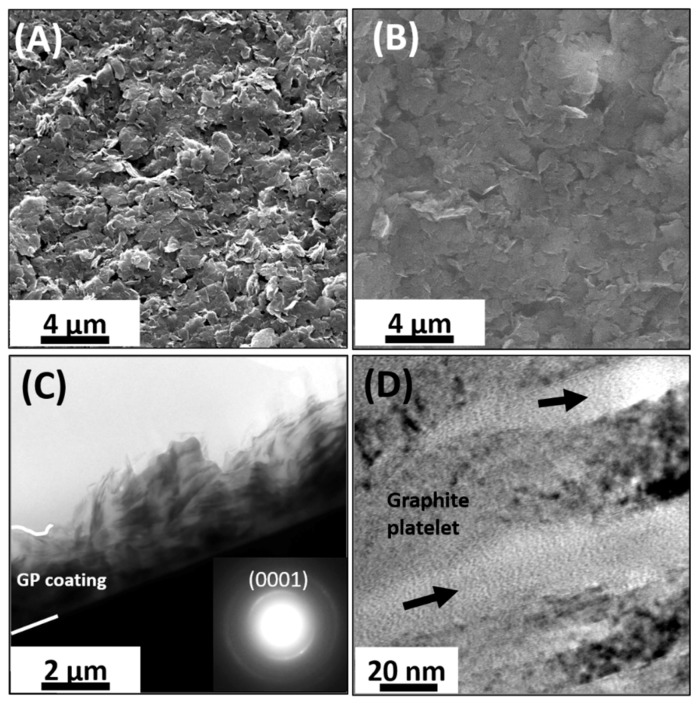
SEM-micrographs of surface topography of GP films deposited on different substrates: low-density polyethylene (LDPE) (**A**) and glass (**B**). Bright field TEM images of cross-sectioned GP films at different magnifications, (**C**,**D**). The inset in (**C**) is the selected area electron diffraction (SAED) pattern of the coating shown in (**C**). Dark arrows in (**D**) evidence the presence of amorphous materials among graphite platelets.

Coating morphology and structure, after spraying Graphit 33 on different substrates, were investigated by SEM and TEM techniques. In particular, Figure 5A,B show the SEM images of the deposited coatings on LDPE and glass substrates, respectively. The coatings on both substrates were quite rough, porous, and were made of small graphite platelets, which covered all substrates without discontinuities. TEM micrographs of a cross-sectioned GP coating are displayed in Figure 5C,D. The coating is rather wrinkled, as shown in Figure 5C, with a thickness ranging from 2.3 to 3.6 µm and a mean value of about 2.5 µm was measured on the large areas. TEM and SEM analyses suggest a surface roughness of 500–1000 nm. The current application of this study deals with macroscopic thermoresistors, therefore, the surface roughness of the platelet films did not present any issue. Indeed, the metal contacts could have an arbitrary size and could be formed by a silver paste coating or by metal sputtering (Au), using a shadow mask. Clearly, in the case of a microscopic device, the roughness of the film could hamper the fabrication of micro-nano patterns, through an advanced lithographic process.

By increasing the magnification (Figure 5D), it is possible to observe that coating is made of overlapping graphite platelets of different thickness, ranging from tens to hundreds of graphene layers. Among the platelets, the polymeric binder visibly generates a lighter contrast typical of amorphous material (see arrows). Although, the platelets assume all orientations locally in the selected area electron diffraction (SAED) pattern of a large part of the coating (shown in the inset of Figure 5C) it could be observed that the ring corresponding to the (0001) atomic planes had a non-uniform intensity that could be correlated to the platelet’s tendency to align, in average, parallel to the interfacial plain.

**Figure 6:**
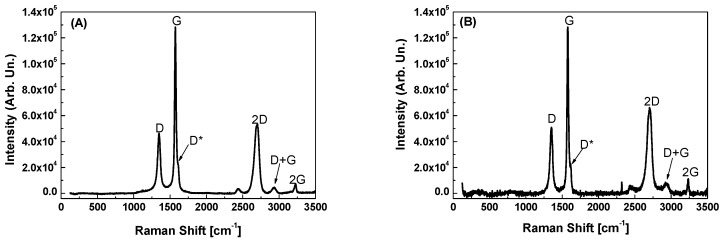
Raman spectra of graphite platelet (GP) films deposited on glass (**A**) and LDPE (**B**).

Further structural characterizations were performed by Raman spectroscopy. The following spectra are related to GP films deposited on glass (Figure 6A) and LDPE (Figure 6B).

Spectra analysis of GP films deposited both on glass and LDPE substrates indicated that the coatings present similar characteristics, with a narrow D peak and a sharp and narrow G peak, right shouldered (D*) due to the presence of defects [25,26,27]. The mean value of the ratio between the intensities of the two peaks, I_d_/I_g_, over the sampled points, was about 0.55 in the case of GP on glass and about 0.80 for GP on LDPE, revealing that the films were made of a good quality graphitic material with a lower presence of defects in the film deposited on glass. Three other peaks could be detected in both spectra at higher wavenumbers (2300 to 3400 cm^−1^)–an intense left shouldered 2D peak (indicating a multilayered structure), a D + G peak, and a 2G peak normal in width and intensity for a graphitic material. No substrate signal was detected because the thickness of the GP film was larger than the penetration length of laser radiation used in the Raman apparatus.

### 3.3. Thermal Properties of Pure LDPE and LDPE Coated by GP Films

**Figure 7:**
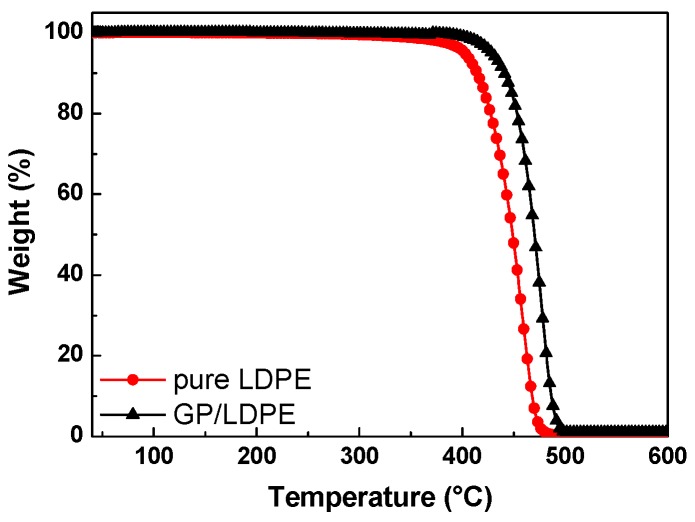
TGA–thermograms of pure LDPE and LDPE coated by GP.

The thermal stability of the pure LDPE and LDPE coated by GP was evaluated by TGA measurements. According to the TGA–thermograms, up to a temperature of ca. 130 °C, both samples were stable since they did not show any weight loss. Furthermore, by comparing the residual mass at 600 °C, which had TGA curves in the 40–600 °C range, it could be seen that the average amount of coating was ca. 1% by weight of the full LDPE/GP system. The graphite coating had the effect of increasing the pure LDPE substrate thermal stability by ca. 22 °C (see Figure 7).

In order to correlate the effects of the thermal expansion of the LDPE substrate with the electrical properties of graphite platelet films, tests of thermal expansion and contraction were carried out on a sample of LDPE coated by GP. In particular, this characterization was carried out between −40 °C and 40 °C, because in this temperature range, LDPE did not show any phase transition such as crystallisation, melting, or glass transition, and the stress–strain response induced by temperature variations was quite reversible [28].

**Figure 8:**
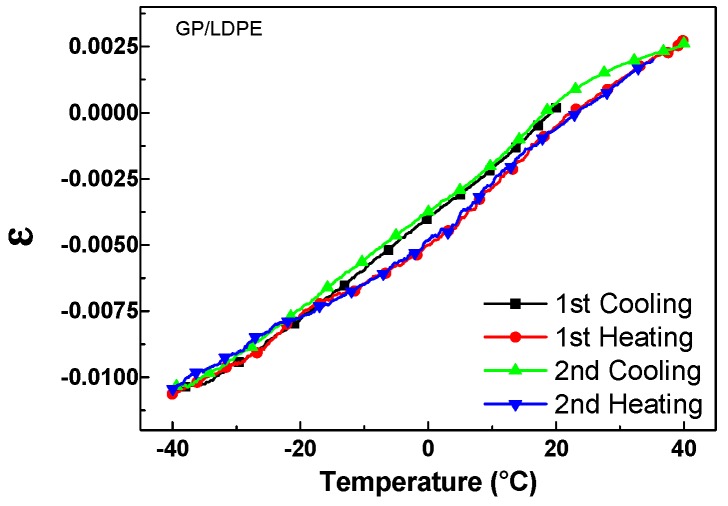
Strain, ε, vs. temperature of the LDPE coated by GP for two consecutive cooling–heating cycles.

The length of the sample, L, was recorded applying a constant force of 0.01 N and varying the temperature in the above range at the rate of 5 °C/min. The strain of the sample, defined as ε = (L − L_0_)/L_0_, where L_0_ is the initial sample length at 20 °C, is plotted in Figure 8, for two consecutive cooling–heating cycles. A small hysteresis was evident between the heating and cooling curves. It could be due to both a thermal relaxation of the LDPE molecules and a small temperature gradient normally present in the furnace during cooling. The coefficient of linear thermal expansion, CTE, of the LDPE coated by the GP sample was calculated as ε/ΔT, where ΔT is the temperature change during the test. CTE value results to be about 1.7 × 10^−4^ °C^−1^ on the investigated temperature range. This value was in good agreement with those reported in the literature for LDPE films (1–2 × 10^−4^ °C^−1^) [29].

### 3.4. Thermoresistive Characterizations of Graphite Platelet Films on Glass and LDPE

**Figure 9:**
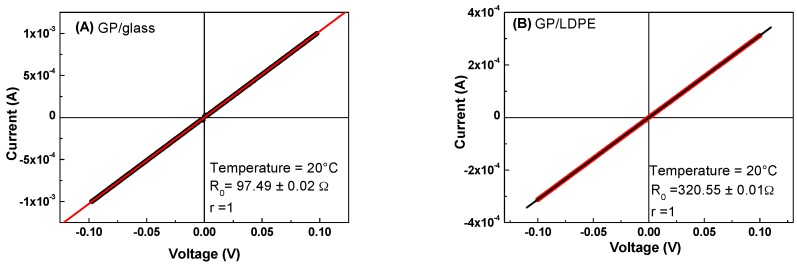
I–V characteristics of the GP films deposited on glass (**A**) and LDPE (**B**) substrates.

All electrical measurements were carried out under vacuum in two probe configuration. The I–V characteristics of the GP films deposited on glass and LDPE were linear, indicating ohmic contacts, as shown in Figure 9.

The resistance values, R_0_, of the samples at the temperature of 20 °C were determined by the fit of the plotted experimental data.

The thermoresistive properties of the GP films were investigated by recording the resistance values, R, of the samples starting from 20 °C and performing the cooling–heating cycles in the −40 to 40 °C range. The high resistance value of GP on LDPE could be due to the larger surface roughness of the polymer substrate and its different chemical nature, compared to the glass ones that determined a greater degree of inhomogeneity, as evidenced by the increased I_d_/I_g_ ratio obtained by Raman analysis.

**Figure 10:**
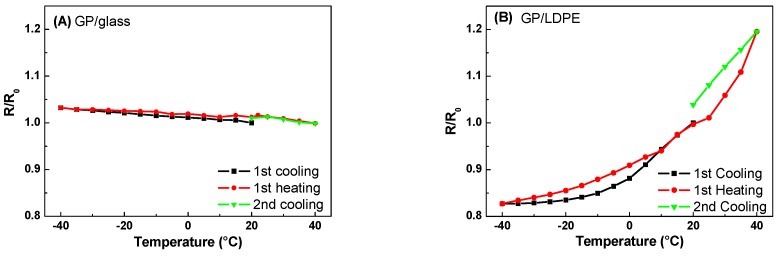
R/R_0_ vs. temperature during the cooling–heating cycles for GP films deposited on glass (**A**) and LDPE (**B**) substrates.

In Figure 10, the R/R_0_ ratios are plotted against temperature for representative samples of GP films deposited on glass and LDPE, respectively. Clearly, different thermoresistive behaviors could be observed in the examined range. Indeed, the resistance of GP film on glass slowly decreases with increasing T, similar to that of graphite [30], while GP on LDPE shows an increase in resistance in the whole range. Thus, thermoresistive properties of GP films strongly depend on the substrates. In fact, TCR defined as:

(1) $$TCR=\frac{1}{R}\frac{dR}{dT}$$

is negative for GP on glass and positive for GP on LDPE.

Taking into account the cooling–heating cycles of Figure 10A,B, TCR varies in the −4 × 10^−4^ to −7 × 10^−4^ °C^−1^ and 4 × 10^−3^ to 8 × 10^−3^ °C^−1^ ranges for GP on glass and LDPE, respectively. Furthermore, a greater reproducibility of the resistance–temperature characteristics in the case of GP film deposited on the glass was observed. On the other hand, the appreciable thermoresistive sensitivity of GP on LDPE made these materials useful for flexible electronic applications, although more work has to be done to reduce the hysteresis during the thermal cycles and obtain a GP material with a more reversible thermoresistive response.

**Figure 11:**
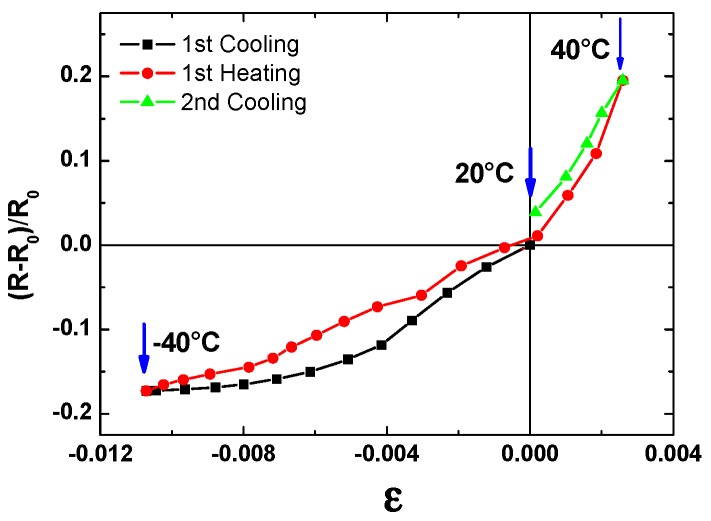
The fractional change of the electrical resistance, (R − R_0_)/R_0_, vs. the strain, ε, of the GP film on the LDPE substrate.

The observed behaviors could be attributed to the different CTE of the substrates. Glass has a CTE (6–9 × 10^−6^ °C^−1^) close to that of graphite (4–8 × 10^−6^ °C^−1^), and therefore, the resistance of the GP film as compared to T, decreases, as in the case of graphite [29]. On the other hand, as reported in Section 3.3, the CTE of the coated LDPE (1.7 × 10^−4^ °C^−1^) is more than one order of magnitude greater than that of graphite, thus, the thermal expansion (contraction) of the polymer substrate could induce strains in the GP film, which tend to increase (decrease) its resistance. For example, in the case of the investigated sample, by comparing the data in Figure 8 and Figure 10B, the fractional change of the electrical resistance, (R − R_0_)/R_0_, of the GP film could be correlated to the strain, ε, of the coated LDPE substrate, as shown in Figure 11.

As can be seen, the slopes of the curves in the −40 to 20 °C range were lower than those in the 20 to 40 °C range. Indeed, below the sample deposition temperature (20 °C), a greater compaction of the platelets enhanced the decrease (increase) in the resistance during heating (cooling), as in the case of graphite. This effect tends to counterbalance the increase (decrease) in resistance due to the expansion (contraction) of the substrate and, therefore, the resulting resistance increases (decreases) more slowly in this temperature range.

Additionally, the larger resistance hysteresis occurring in the GP film on LDPE during the cooling–heating cycles (Figure 10B) could be attributed to the greater CTE of the LDPE, compared to that of glass. Indeed, the platelets deposited on LDPE were subjected to a greater mobility due to the strains of this polymer substrate and the occurrence of possible nano/micro fractures in the films that could cause a different assembly of the platelets when the sample passed again for the same temperature during a thermal cycle, leading to a different value of its initial resistance.

## 4. Conclusions

It was observed that large-area conductive thin films could be produced by spraying Graphit 33 lacquer on glass and LDPE substrates. Raman spectra analysis revealed that the graphitic material deposited on both substrates was of good quality. According to the morphological and structural investigations by SEM and TEM, the films consist of overlapped graphite platelets that cover the surfaces of the substrates, mostly in a coplanar manner. It was found that the resistance of the film as a function of temperature in the −40 to 40 °C range decreased if the substrate was glass and increased in the case of the LDPE substrate. Therefore, the temperature coefficient of resistance changed from negative to positive values, respectively. It was demonstrated that the different thermoresistive properties of the GP films depend on the thermal expansion characteristics of the substrates on which they have been deposited. The appreciable thermoresistive sensitivity of GP films on LDPE makes these structures promising for applications in plastic electronics, however, more work has to be carried out to reduce the hysteresis observed during the thermal cycles to obtain a GP material with more reproducible thermoresistive properties.
